# The tumour suppressor brain tumour (Brat) regulates linker histone dBigH1 expression in the *Drosophila* female germline and the early embryo

**DOI:** 10.1098/rsob.200408

**Published:** 2021-05-05

**Authors:** Paula Climent-Cantó, Albert Carbonell, Srividya Tamirisa, Laszlo Henn, Salvador Pérez-Montero, Imre M. Boros, Fernando Azorín

**Affiliations:** ^1^ Institute of Molecular Biology of Barcelona, CSIC, Barcelona 08028, Spain; ^2^ Institute for Research in Biomedicine, IRB Barcelona, The Barcelona Institute for Science and Technology, Barcelona 08028, Spain; ^3^ Institute of Biochemistry, Biological Research Centre of Szeged, Szeged 6726, Hungary; ^4^ Department of Biochemistry and Molecular Biology, Faculty of Science and Informatics, University of Szeged, Szeged 6726, Hungary

**Keywords:** linker histone H1, dBigH1, Brat, oogenesis, embryogenesis, *Drosophila*

## Abstract

Linker histones H1 are essential chromatin components that exist as multiple developmentally regulated variants. In metazoans, specific H1s are expressed during germline development in a tightly regulated manner. However, the mechanisms governing their stage-dependent expression are poorly understood. Here, we address this question in *Drosophila*, which encodes for a single germline-specific dBigH1 linker histone. We show that during female germline lineage differentiation, dBigH1 is expressed in germ stem cells and cystoblasts, becomes silenced during transit-amplifying (TA) cystocytes divisions to resume expression after proliferation stops and differentiation starts, when it progressively accumulates in the oocyte. We find that dBigH1 silencing during TA divisions is post-transcriptional and depends on the tumour suppressor Brain tumour (Brat), an essential RNA-binding protein that regulates mRNA translation and stability. Like other oocyte-specific variants, dBigH1 is maternally expressed during early embryogenesis until it is replaced by somatic dH1 at the maternal-to-zygotic transition (MZT). Brat also mediates dBigH1 silencing at MZT. Finally, we discuss the situation in testes, where Brat is not expressed, but dBigH1 is translationally silenced too.

## Graphical abstract

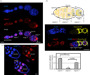

## 1. Introduction

Linker histones H1 constitute a conserved family of chromosomal proteins that bind nucleosomes and play central roles in the regulation of chromatin structure and function. Metazoan species usually contain multiple H1 variants that show differential patterns of expression during development and differentiation. In this regard, a conserved feature in metazoans is the presence of germline-specific variants that replace somatic H1s in germ cells (reviewed in [1]). In many cases, female- and male-specific variants have been described. For instance, mammals usually contain three testis-specific H1s (H1T, HILS1 and H1T2) [2–7] and one female-specific variant (H1oo) [8]. The presence of female- and male-specific H1s has also been reported in *Xenopus* (B4 and H1fx) [9,10] and the sea urchin (Cs-H1 and SpH1) [11–13], while in *Caenorhabditis elegans*, the situation is more complex since the H1.1/HIS-24 variant is present in both the female and male germline, but it is also detected in somatic cells [14,15]. Female-specific variants have also been described in the zebrafish (H1M) [16,17] and echiura (H1M) [18]. Instead, *Drosophila* encodes for a single germline-specific linker histone, dBigH1, that is expressed in both the male and the female germline [19]. Female-specific H1s usually persist during embryo development until the zygotic genome is activated at MZT [8,10–12,16–21].

In general, the patterns of expression of germline H1s are tightly regulated during lineage differentiation. Testis-specific variants are generally detected only after spermatogonia stop proliferation and differentiate to spermatocytes. In mammals, H1T is the first variant to be expressed in meiotic spermatocytes and, depending on the species, is also detected during spermatids differentiation [22–25]. On the other hand, expression of the other two mammalian male variants HILS1 and H1T2 is restricted to spermatids [4–7]. A similar situation is observed in *Drosophila*, where dBigH1 is detected in spermatocytes, but not in the proliferating spermatogonia and upon spermatids differentiation [19]. dBigH1 is also detected in the male germ stem cell (GSC). Regarding female-specific H1s, their expression is mostly restricted to the oocyte and the early stages of embryo development. In humans and mice, H1oo expression is restricted to the growing/maturing oocyte entering meiosis and, after fertilization, it rapidly decays during the first mitotic divisions, becoming undetectable at the 2–4 cells blastula stage when it is replaced by the somatic H1 variants [8,21,26,27]. In the sea urchin, the female-specific Cs-H1 variant is also replaced by somatic H1s at early cleavage stages [11,12]. However, the female-specific variants of *Drosophila* (dBigH1), zebrafish (H1M) and *Xenopus* (B4) persist longer during embryo development, being replaced by the somatic H1s only after 14, 10 and 13 cleavages, respectively [10,16,17,19]. Translational regulatory mechanisms appear to play a central role in the regulation of germline H1s expression. In *Xenopus*, translation of B4 mRNA is regulated by CPEBs proteins that bind at the 3′UTR and, upon phosphorylation, promote polyadenylation [28–30]. Mammalian H1oo mRNA also contains several functional CPEs at the 3′UTR [31,32]. In addition, in testes, several 5'UTR regulatory elements regulate HILS1 expression [4,33], and in *Drosophila* the translational repressor Bam is required to silence dBigH1 expression during spermatogonia proliferation [34]. However, little else is known about the mechanisms that govern stage-specific expression of germline H1s. Here, we address this question in the *Drosophila* female germline.

In *Drosophila*, the early stages of gametogenesis share remarkable similarities in females and males (reviewed in [35–39]). In both ovaries and testes, GSCs localize anterior, anchored to a niche of somatic cells, and divide asymmetrically for self-renewal and to produce daughter progenitor cells (cystoblasts (CBs) in females and gonioblasts (GBs) in males), which start the complex differentiation programme that, ultimately, leads to the production of functional gametes. Daughter cells undergo four successive rounds of transit-amplifying (TA) divisions with incomplete cytokinesis to produce a cyst of 16 sister germ cells (GCs) (spermatogonia in males and cystocytes in females) that remain interconnected and are surrounded by a somatic cells layer. Then, the pathways diverge; female cysts develop to produce a single egg, whereas male cysts differentiate to spermatocytes and undergo two meiotic divisions to produce 64 spermatids that develop to mature sperm cells. Our results show that, similar to males, dBigH1 is expressed in the female GSCs and CBs, is silenced in the proliferating TA cystocytes to resume expression upon oocyte differentiation. We report that dBigH1 silencing in cystocytes depends on the tumour suppressor Brain tumour (Brat), a post-transcriptional regulator that is expressed in cystocytes and represses translation of GSC maintenance factors [40,41]. We also show that, during embryogenesis, Brat silences dBigH1 expression at MZT. Altogether these results unveil the importance of post-transcriptional regulation in setting the patterns of expression of germline-specific H1 variants.

## 2. Material and methods

### 2.1. Fly stocks and genetic procedures

*w^1118^*, *nos-Gal4::VP16* [42] and *Df(2 L)TE37C-7* were obtained from Bloomington *Drosophila* Stock Center (BDSC). *brat^RNAi^* and *bam^RNAi^* correspond to stocks 28 590 (y[1] v[1]; P{y[+t7.7] v[+t1.8] = TRiP.HM05078}attP2) and 33 631 (y[1] v[1]; P{y[+t7.7] v[+t1.8] = TRiP.HMS00029}attP2) from BDSC, respectively. *dBigH1^NTSOP^* CRISPR/CAS9 mutant is described in [43]. *bamP-bam::GFP* and *bamP-*GFP [44] were a gift from Dr M. Buszczak. *brat^K06028^* was a gift from Dr J. Knoblich. *vasa*-EGFP construct [45] was a gift from Dr A. Nakamura. Transgenic lines carrying the various constructs described in figures 4 and 7 and electronic supplementary material, figure S4 were obtained by specific site-directed integration into ZH-86Fb and ZH-58A att line [46]. All *Drosophila* stocks were maintained at 25°C on standard media. For RNAi knockdown, crosses were set up at 25°C.

### 2.2. Antibodies

Rabbit polyclonal αdBigH1 is described in [19]. Rat polyclonal αdBigH1 was raised as described in [19]. Rabbit αdH1 was a gift from Dr J. Kadonaga and is described in [47]. Guinea pig polyclonal αTj was a gift from Dr D. Godt and is described in [48]. Rabbit αBrat was a gift from Dr J. Knoblich and is described in [49]. All other antibodies used in these experiments were commercially available: mouse monoclonal αadd (DSHB, 1B1), rat monoclonal αHA (Sigma, 3F10), mouse monoclonal αFasciclin III (DSHB, 7G10) and rat monoclonal αVasa (DSHB, 1ea).

### 2.3. Immunostaining experiments

**Figure 1.**
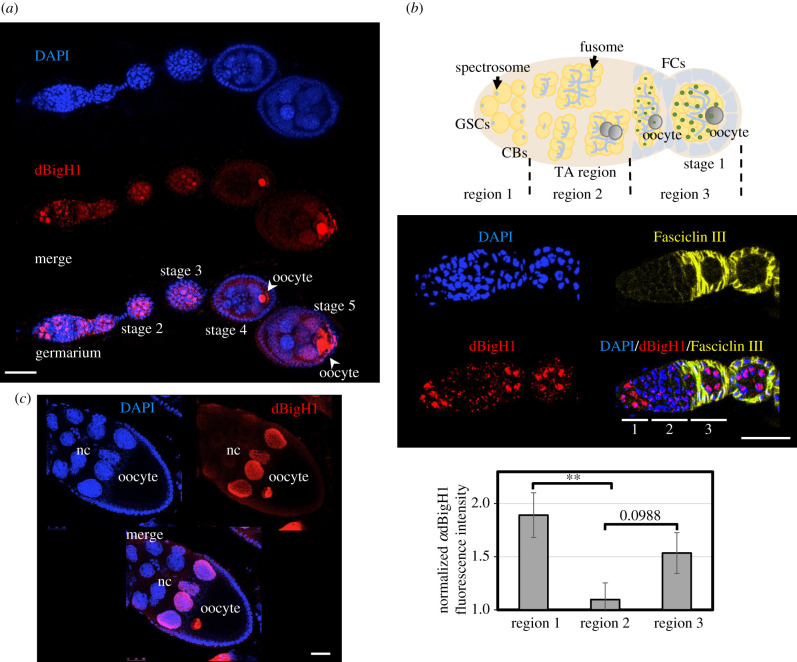
The pattern of dBigH1 expression in *Drosophila* ovaries. (*a*) Immunostaining with αdBigH1 antibodies (in red) of an ovariole. The germarium and different stages of egg chamber development are indicated. The position of the oocyte is also indicated. DNA was stained with DAPI (in blue). Scale bar corresponds to 25 µm. (*b*) Top: schematic of the germarium. Centre: immunostainings with αdBigH1 (in red) and αFasciclin III antibodies (in yellow), which label the somatic follicle cells (FC) surrounding the 16 cells cysts and the emerging egg chambers. Regions 1, 2 and 3 of the germarium are indicated. DNA was stained with DAPI (in blue). Scale bar corresponds to 25 µm. Quantitative analysis is shown at the bottom, where the intensity of αdBigH1 immunostaining at regions 1, 2 and 3 is presented. (*N* = 3; *n* = 11; error bars are s.e.m.; two-tailed *t*-Student, *p*-value: ** < 0.01.) (*c*) Immunostaining with αdBigH1 antibodies (in red) of a developing egg chamber (stage 10). The nurse cells (nc) and oocyte are indicated. DNA was stained with DAPI (in blue). Scale bar corresponds to 30 µm. See also electronic supplementary material, figures S1–S3.

Ovaries and testes were dissected in PBS, fixed in PBS with 4% paraformaldehyde for 20 min, and then washed with PBS three times for 10 min each. The samples were first incubated in blocking solution, 2% bovine serum albumin diluted in PBT (PBS and 0.3%Triton X-100), for 1 h and then with the appropriate primary antibodies diluted in blocking solution at 4°C overnight. The samples were washed with PBT three times for 10 min each and then incubated with blocking solution for 30 min. Then, samples were incubated with the appropriate secondary antibodies at 25°C for 2 h and washed with PBT three times for 10 min. Samples were mounted in Mowiol (Calbiochem-Navabiochem) containing 0.2 ng µl^−1^ DAPI (Sigma) and visualized in a confocal microscope (Leica TCS SP2-AOBS). Quantitative analyses presented in figures 1*b* and 4*d* were performed using ImageJ. In each germarium, DAPI was used to define the nuclear area of three different cells from each region (1–3). Three additional ROIs (same area and non-nuclear) were also defined as background. In figure 1*b*, the nuclear signal of αdBigH1 was determined using the Gray mean value, and the average signal in each region was normalized against the average background signal. In figure 4*d*, the GFP signal was determined using the same method, but normalization was performed using the average signal of region 2, since the background levels of direct fluorescence were extremely low.

Embryos were dechorionated in bleach and fixed for 25 min in 1 : 1 solution of formaldehyde and heptane. Embryos were devitellinized in methanol followed by rehydration with PBT and blocking in PBT–BSA (2%). Samples were incubated with primary antibodies diluted in PBT–BSA at 4°C overnight. After washing three times with PBT, embryos were incubated with secondary antibody and stained with DAPI at room temperature for 2 h followed by three washes in PBT. Embryos were mounted in vectashield (Vector labs) and imaged using Zeiss LSM 780 confocal microscope.

Primary antibodies used for immunostaining were: αdBigH1 (1 : 400), αdH1 (1 : 4000), αadd (1 : 100), αHA (1 : 200), αFasciclin III (1 : 20), αVasa (1 : 300), αTj (1 : 5000) and αBrat (1 : 100). Secondary antibodies conjugated with Cy2 and Cy3 (Jackson Immuno Research) were used at a 1 : 300 dilution.

### 2.4. RT–qPCR analysis

For qRT–PCR experiments, RNA was prepared from embryos using trizol reagent and purified using qiagen RNeasy mini kit following the manufacturer's instructions. Total RNA (1 µg) was used for complementary DNA (cDNA) synthesis. Reverse transcription was performed using oligo dT supplied in the kit. qRT–PCRs were run in triplicate in two independent experiments. Expression data were normalized to *Act5C* and analysed using the ΔΔCt method. Primers used were: dBigH1fw 5′-AATATGGGCGAAGAAGAGGA-3′, dBigH1rv 5′-GAGATTATCTGTCTCGACCTC-3′, Act5cfw 5′-CACCAAATCTTACAAAATGTGTGAC-3′ and Act5crv 5′-CATCGTCTCCGGCAAATC-3′.

## 3. Results

### 3.1. dBigH1 expression is silenced during TA cystocytes divisions

**Figure 2.**
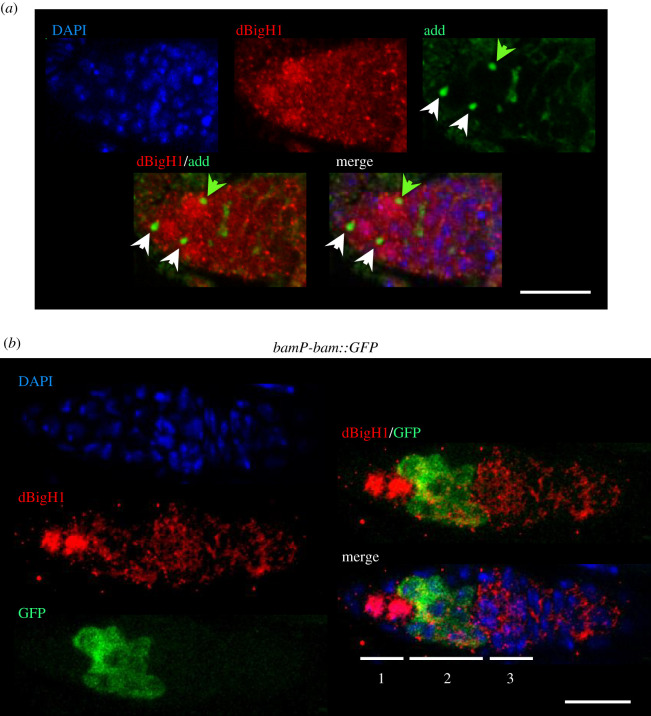
dBigH1 expression is silenced in TA cystocytes. (*a*) Immunostainings with αdBigH1 (in red) and αadd antibodies (in green), which label the spectrosome. Only the tip region of the germarium, which contains the GSCs and CBs, is shown. Arrows indicate spectrosomes occupying an anterior (white) or a posterior (green) position. DNA was stained with DAPI (in blue). Scale bar corresponds to 15 µm. (*b*) The pattern of expression of a *bamP-bam::GFP* reporter construct. dBigH1 was immunostained with αdBigH1 antibodies (in red). GFP was direct fluorescence. Regions 1, 2 and 3 of the germarium are indicated. DNA was stained with DAPI (in blue). Scale bars correspond to 15 µm.

**Figure 3.**
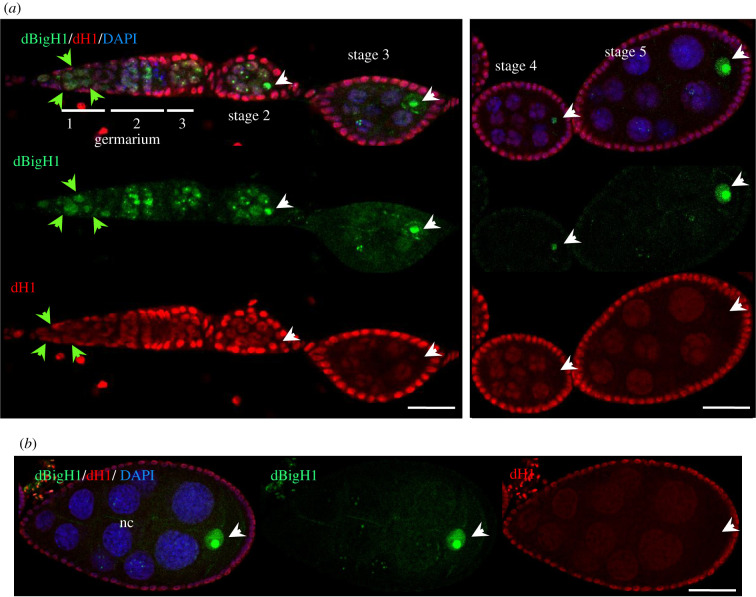
The pattern of expression of somatic dH1 in *Drosophila* ovaries. (*a*) Immunostainings with αdBigH1 (in green) and αdH1 (in red). The germarium and different stages of egg chamber development are indicated. Arrows indicate GSCs/CBs (green) and the oocyte nucleus (white). DNA was stained with DAPI (in blue). Scale bar corresponds to 25 µm. (*b*) Immunostainings with αdBigH1 (in green) and αdH1 (in red) of an egg chamber at developmental stage 8. The nurse cells (nc) and oocyte nucleus (arrow) are indicated. DNA was stained with DAPI (in blue). Scale bars correspond to 25 μm.

Immunofluorescence (IF) experiments detected dBigH1 expression throughout oogenesis, from the germarium to the latest stages of egg chamber development (figure 1*a*). Female germline lineage differentiation begins at the germarium that, at the most anterior part, contains 2–3 female GSCs and the daughter CBs (region 1), which divide to generate developing cysts of increasing number of cystocytes (region 2). Then, at the 16-cell stage, cysts are surrounded by somatic epithelial follicle cells (FC), and bud off the germarium as individual egg chambers (region 3) [50] (figure 1*b*, top). In the germarium, intense nuclear αdBigH1 immunostaining was detected in region 1, being highly reduced to background levels during cystocytes proliferation in region 2, to reappear again in region 3 (figure 1*b*, centre and bottom). Cells showing αdBigH1 immunostaining were positive for vasa, a specific germline marker [51,52], and negative for Traffic jam (Tj), a marker of somatic cells [48] (electronic supplementary material, figure S1), suggesting that dBigH1 expression was restricted to germ cells. We also analysed whether, in region 1, dBigH1 was expressed in both GSCs and CBs. For this purpose, we performed co-immunostaining experiments with αadd antibodies, which mark the spectrosome, a cytoskeleton structure that occupies an anterior position in GSCs, moves posterior in CBs and, later, grows and branches out to form the fusome that keeps cysts cells interconnected [53,54] (figure 1*b*, top). In region 1, we detected nuclear αdBigH1 immunostaining in cells with anterior as well as posterior spectrosomes (figure 2*a*), indicating that dBigH1 was expressed in both GSCs and CBs. In addition, silencing of dBigH1 expression during cystocytes proliferation was confirmed in flies carrying a *bamP*-*bam::GFP* construct, which is specifically expressed in cystocytes [44], since nuclear αdBigH1 immunostaining was not detected in cells expressing the reporter (figure 2*b*). After cystocytes stop proliferation and start to differentiate, dBigH1 expression resumed. In the budding cysts (region 3 of the germarium) and the early-developed egg chambers (stage 2), all nuclei were positive for αdBigH1 (figure 1*a*). Later, from stage 3 on, αdBigH1 immunostaining was progressively constrained to the oocyte nucleus (figure 1*a,c*), where it largely overlapped with DAPI at the condensed chromatin of the karyosome (electronic supplementary material, figure S2). A weak signal could also be detected in the nucleoplasm (electronic supplementary material, figure S2B; see also figure 3*b*), suggesting that a minor fraction of dBigH1 stays unbound and free in the nucleoplasm. At late developmental stages, αdBigH1 immunostaining was also detected in nuclei of the nurse cells (nc) proximal to the oocyte (figure 1*a*,*c*). This pattern of expression is very unusual and, interestingly, takes place around the stage when nc begin dumping of their content into the oocyte and, ultimately, die. In this regard, nc proximal to the oocyte are first in undergoing dumping. Though highly speculative, dBigH1 might regulate transcriptional activity in these cells during dumping. Of note, nuclear αdBigH1 immunostaining of germ cells was abolished in a null *dBigH1^NSTOP^* CRISPR/CAS9 mutant [43], showing its specificity (electronic supplementary material, figure S3). By contrast, background αdBigH1 immunostaining observed in the cytoplasm and somatic FC was also detected in the null *dBigH1^NSTOP^* mutant, indicating it was unspecific (electronic supplementary material, figure S3).

We also analysed the pattern of expression in ovaries of the single somatic linker histone of *Drosophila* dH1 [55–57]. In addition to the somatic FC cells, which showed strong αdH1 immunostaining, we also detected dH1 expression in germline cells (figure 3*a*). In the germarium, αdH1 signal was detected in the GSCs/CBs, which express dBigH1, as well as in cystocytes, which lack dBigH1 (figure 3*a*). In the budding cysts and stage 2 egg chambers, dH1 expression was detected in both the nc and the oocyte that also contained dBigH1 (figure 3*a*). Later, when dBigH1 starts to accumulate in the oocyte (stages 3–5), dH1 expression decayed in the oocyte, becoming undetectable at stage 5 (figure 3*a*), while it was still detected in the nc (figure 3*a*). In the nc, dH1 expression also decreased upon development to almost undetectable levels (figure 3*b*).

### 3.2. Silencing of dBigH1 in cystocytes is post-transcriptionally regulated

**Figure 4.**
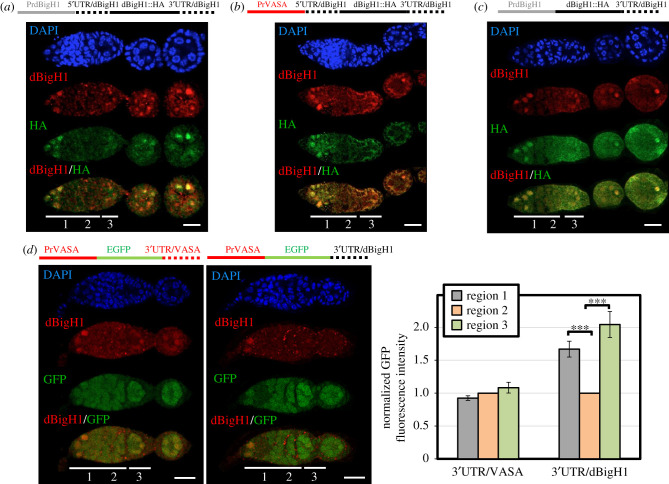
dBigH1 silencing in cystocytes depends on the 3′UTR. (*a*–*c*) The patterns of expression of ectopic dBigH1::HA constructs carrying the indicated *cis*-regulatory elements are presented. Immunostainings with αdBigH1 (in red) and αHA antibodies (in green) are shown. Regions 1, 2 and 3 of the germarium are indicated. DNA was stained with DAPI (in blue). Scales bar correspond to 15 µm. (*d*) The patterns of expression of EGFP constructs carrying the indicated *cis*-regulatory elements are presented. GFP was direct fluorescence. DNA was stained with DAPI (in blue). Scale bars correspond to 15 µm. Quantitative analysis is shown in the right, where the intensity of GFP fluorescence in regions 1, 2 and 3 is presented for the indicated constructs. (*N* = 2; *n* = 25; error bars are s.e.m.; two-tailed *t*-Student, *p*-value: ***<0.001.) See also electronic supplementary material, figure S4A.

Results reported above suggest that dBigH1 expression is tightly regulated during early oogenesis, being silenced in proliferating cystocytes. This regulation is mainly post-transcriptional since expression of a ectopic dBigH1::HA construct, which carries the *dBigH1* regulatory elements and largely mimics expression of endogenous dBigH1 (figure 4*a*), was not substantially altered when the *dBigH1* promoter was replaced by the germline-specific *vasa* promoter, which is ubiquitously active in germline cells [45] (figure 4*b*) (see also figure 4*d*). We also observed that the deletion of the *dBigH1* 5′UTR had no major effect on the pattern of dBigH1::HA expression in ovaries (figure 4*c*), suggesting that the *dBigH1* 3′UTR is sufficient to silence dBigH1 expression in cystocytes. In agreement, we observed that the 3′UTR of *dBigH1* silenced expression in cystocytes of a ubiquitously active *vasa*-EGFP reporter [45] (figure 4*d*). It must be noted that these ectopic dBigH1::HA constructs did not fully recapitulate dBigH1 silencing since we detected dBigH1::HA expression in cystocytes in approximately 25% of germaria. Noteworthy, the proportion of germaria showing ectopic dBigH1::HA expression in cystocytes tended to increase when the *dBigH1* 3′UTR was replaced by that of *vasa* (Fisher test, *p*-value: 0.227) (electronic supplementary material, figure S4A). Altogether these results suggest that elements within the *dBigH1* 3′UTR mediate post-transcriptional silencing in cystocytes.

### 3.3. Brat regulates dBigH1 silencing in cystocytes

**Figure 5.**
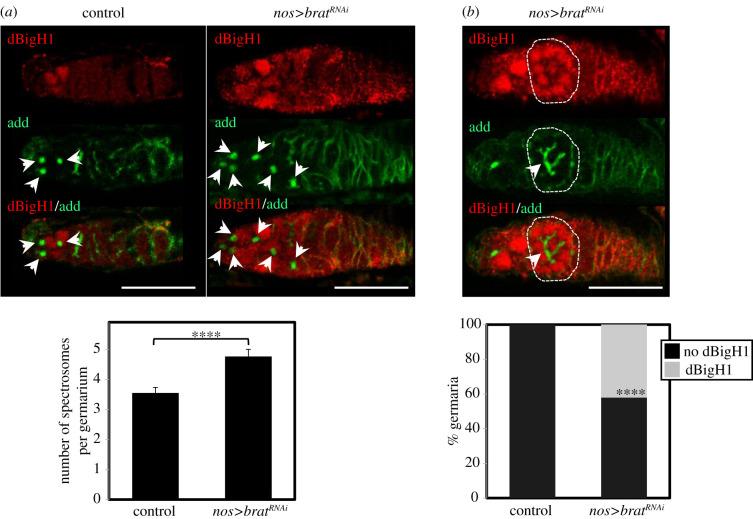
dBigH1 silencing in cystocytes depends on Brat. (*a*) Top: immunostainings with αdBigH1 (in red) and αadd antibodies (in green), which label the spectrosome, of germaria from control wt and *brat^RNAi^* flies in which Brat depletion was induced with a *nos-GAL4::VP16* driver. Only the tip region of the germaria, which contains the GSCs and CBs, is shown. Arrows indicate spectrosomes. DNA was stained with DAPI (in blue). Scale bars correspond to 25 µm. Quantification of the results is shown below, where the number of spectrosomes per germarium is presented (*N* = 2; *n* > 33; error bars are s.d.; two-tailed *t*-Student, *p*-value: ****<0.0001). (*b*) Top: immunostainings with αdBigH1 (in red) and αadd antibodies (in green), which label the fusome, of a germarium from *brat^RNAi^* flies in which Brat depletion was induced with a *nos-GAL4::VP16* driver. A developing cyst showing dBigH1 expression is indicated by the circle. DNA was stained with DAPI (in blue). Scale bar corresponds to 25 µm. Quantification of the results is shown below, where the proportion of germaria containing αdBigH1-positive cysts is presented (*N* = 2; *n* = 38; error bars are s.d.; Fisher test, *p*-value: ****<0.0001). See also electronic supplementary material, figures S5 and S6.

We noted that the *dBigH1* 3'UTR sequence contains two consensus binding sites for Brat [58] (electronic supplementary material, figure S5A), an important post-transcriptional regulator that is expressed in cystocytes (electronic supplementary material, figure S6) and represses translation of stem cell maintenance factors, promoting differentiation [40,41]. Interestingly, RNA immunoprecipitation experiments (RIP-Chip) performed in embryos showed that Brat interacts with the *dBigH1* mRNA [58]. Thus, we tested the possibility that Brat is involved in silencing dBigH1 expression in cystocytes. For this purpose, we performed RNAi-mediated depletion of Brat in ovaries using a nos-*GAL4* driver that is specifically expressed in the germline [42]. We observed that, in agreement with its role in promoting GSCs differentiation, Brat depletion increased the number of cells in which spectrosome structures were detected (figure 5*a*), suggesting an accumulation of GSCs/CBs. In addition, approximately 40% of germaria showed detectable levels of dBigH1 expression in cyst cells interconnected by branched fusomes (figure 5*b*), suggesting that Brat is required to silence dBigH1 expression in cystocytes.

**Figure 6.**
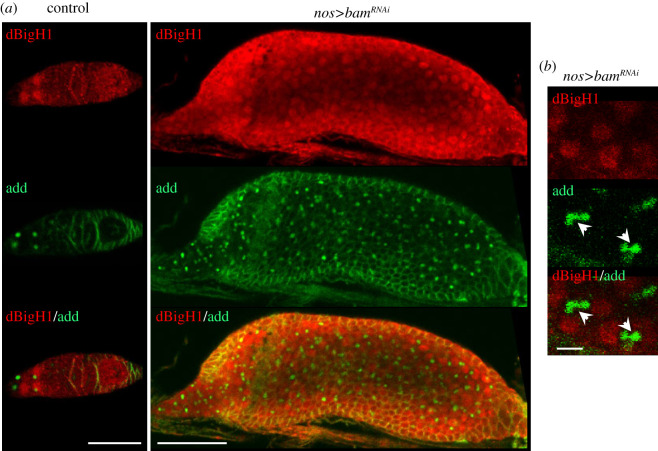
The contribution of Bam to dBigH1 silencing in cystocytes. (*a*) Immunostainings with αdBigH1 (in red) and αadd antibodies (in green) of germaria from control wt and *bam^RNAi^* flies in which Bam depletion was induced with a *nos-GAL4::VP16* driver. Arrows indicate spectrosomes. Scale bars correspond to 25 µm. (*b*) Enlarged image of immunostainings with αdBigH1 (in red) and αadd antibodies (in green) of early-developed cysts showing dBigH1 expression from *bam^RNAi^* flies in which Bam depletion was induced with a *nos-GAL4::VP16* driver. Arrows indicate growing fusomes. Scale bar corresponds to 5 µm.

**Figure 7.**
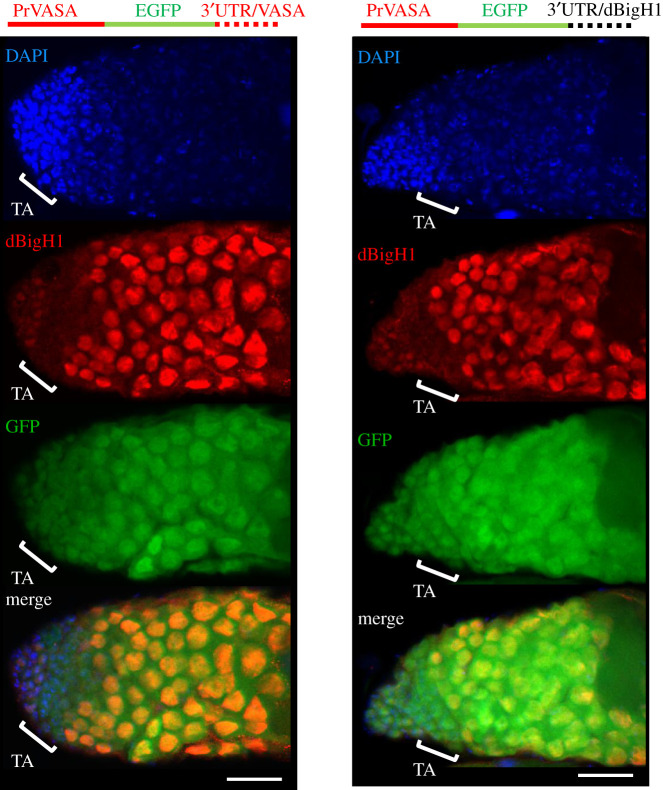
The *dBigH1 3′UTR* does not silence gene expression in male TA-spermatogonia. The patterns of expression of EGFP constructs carrying the indicated *cis*-regulatory elements are presented. The position of the TA region is indicated. GFP was direct fluorescence. DNA was stained with DAPI (in blue). Scale bars correspond to 25 µm. See also electronic supplementary material, figure S4B.

It was shown earlier that, in testes, dBigH1 expression is post-transcriptionally silenced during TA spermatogonial divisions by bag-of-marbles (Bam) [34]. Bam is also expressed in ovaries [59,60], where it represses translation of GSC maintenance factors and induces differentiation [61–63]. In this regard, it is known that Bam is required for Brat expression in cystocytes since it represses the GSC maintenance factor nanos (nos) [63,64] that, in its turn, represses Brat [40]. Thus, we anticipated that Bam would also regulated dBigH1 expression in cystocytes. We observed that the depletion of Bam in ovaries blocked early differentiation, giving rise to tumorous germaria that contained a large number of undifferentiated GSCs/CBs expressing dBigH1 (figure 6*a*). This strong phenotype, which was reported earlier in other Bam loss-of-function (LOF) mutations [59,65,66], made it challenging to determine the contribution of Bam to dBigH1 silencing in cystocytes. However, among the large number of GSCs/CBs observed upon Bam depletion, we detected dBigH1 expression in some early-developed cysts containing fusome-interconnected cells (figure 6*b*). These results suggest that, like in spermatogonia, Bam also regulates silencing of dBigH1 expression in cystocytes. Besides these similarities, the regulation of dBigH1 silencing in testes and ovaries shows some important differences since, in contrast with what was observed in ovaries, the *dBigH1* 3′UTR was not capable of silencing expression of *vasa*-EGFP in testes (figure 7) and replacement of the *dBigH1* 3′UTR by the *vasa* 3′UTR did not affect silencing in spermatogonia of an ectopic dBigH1::HA construct (electronic supplementary material, figure S4B). In this regard, we considered the possibility that alternative polyadenylation events could give rise to different 3′UTRs in testes and ovaries. However, RACE experiments showed the same *dBigH1* 3′UTR in testes, ovaries and embryos (electronic supplementary material, figure S5A).

### 3.4. Brat also regulates dBigH1 silencing at MZT

**Figure 8.**
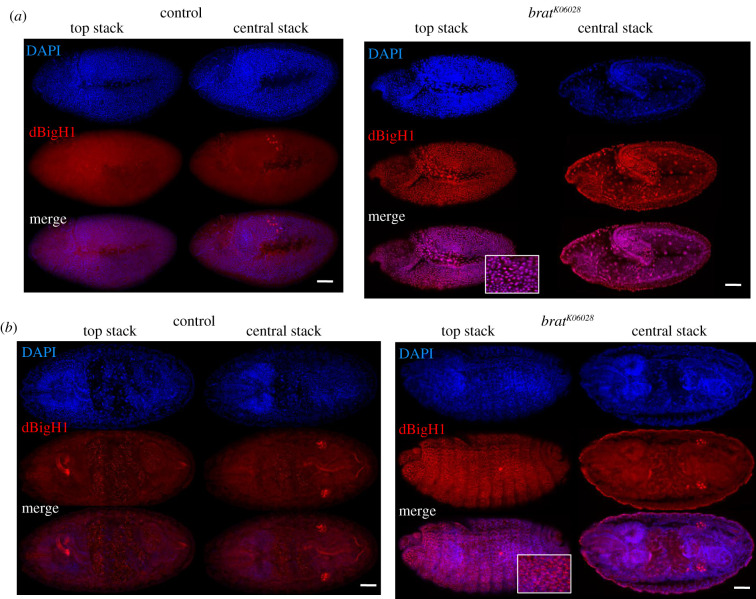
Brat regulates dBigH1 silencing in embryos. Immunostainings with αdBigH1 antibodies (in red) of control wt and homozygous *brat^K06028^* embryos at gastrula stages 9 (*a*) and 15 (*b*). Top and central stacks are presented. DNA was stained with DAPI (in blue). Scale bars correspond to 50 µm. For top stacks of homozygous *brat^K06028^* embryos, enlarged views of merge images showing co-localization of αdBigH1 signal with DAPI are also presented. See also electronic supplementary material, figures S5, S7 and S8.

**Figure 9.**
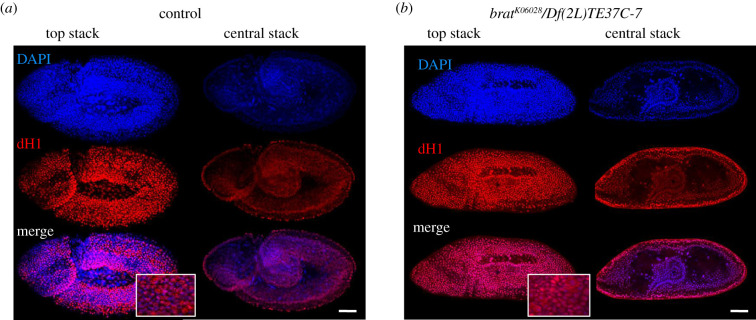
Sustained dBigH1 expression at gastrula stages does not affect dH1 expression. Immunostainings with αdH1 antibodies (in red) of control wt and trans-heterozygous *brat^K06028^*/*Df(2 L)TE37C-7* embryos at gastrula stages 9 (*a*) and 15 (*b*). Top and central stacks are presented. DNA was stained with DAPI (in blue). Scale bars correspond to 50 µm. For top stacks, enlarged views of merge images are also presented.

It has been shown that Brat, which is maternally expressed during early embryogenesis, interacts with and silences a large subset of maternal mRNAs at MZT, among which the *dBigH1* mRNA was identified [58,67]. Thus, we tested the possibility that Brat also silences dBigH1 expression at MZT. For this purpose, we took advantage of the LOF mutation *brat^K06028^*, a recessive lethal P-element insertion allele that shows some defects in abdominal embryo segmentation and induces tumorous overgrowth in larval brains, where Brat is highly expressed [68,69]. Despite these defects, homozygous *brat^K06028^* mutants progress relatively normal through embryogenesis and larval development [58,67,69,70]. We observed that, like in control wild-type embryos, dBigH1 was ubiquitously expressed in homozygous *brat^K06028^* embryos throughout blastoderm stages (electronic supplementary material, figure S7). However, while in control embryos, dBigH1 expression was constrained to the primordial germ cells (PGC) at gastrula stages (figure 8*a*,*b*, left panels), intense αdBigH1 immunostaining was detected in somatic cells in 46% (*N* = 120) of homozygous *brat^K06028^* gastrula (figure 8*a*,*b*, right panels). Similar results were obtained in trans-heterozygous *brat^K06028^/Df(2 L)TE37C-7* embryos, which carried the *brat^K06028^* mutation over the *Df(2 L)TE37C-7* deficiency that uncovers *Brat* (58%, *N* = 108) (electronic supplementary material, figure S8). In addition to its role in translational regulation, Brat has been shown to regulate the stability of a subset of maternal transcripts at MZT, including the *dBigH1* mRNA [58]. RT–qPCR experiments detected increased *dBigH1* mRNA levels in *brat^K06028^* mutant embryos (electronic supplementary material, figure S5B), confirming the contribution of Brat to *dBigH1* mRNA degradation/decay at MZT. Notably, in *brat^K06028^* mutants, intense αdH1 immunostaining was detected at gastrula stages indicating that, under these conditions, dBigH1 and dH1 are both expressed (figure 9).

## 4. Discussion

Results reported here and elsewhere [34] show that the patterns of dBigH1 expression during the early stages of oogenesis and spermatogenesis are remarkably similar. In both cases, dBigH1 is expressed in GSCs and daughter progenitor cells, is silenced during TA divisions to resume expression when proliferation stops and differentiation begins. Moreover, translational regulation accounts for silencing of dBigH1 expression during TA divisions in both ovaries and testes. However, the actual mechanisms involved show some important differences. Our results suggest that, in ovaries, Brat mediates translational dBigH1 silencing in cystocytes by directly binding the *dBigH1* 3′UTR, which contains two Brat-binding sites. However, in testes, the situation must be different since Brat is not significantly expressed (see FlyAtlas and modENCODE tissue expression data in Flybase (https://flybase.org/reports/FBgn0010300)). In good agreement, the *dBigH1* 3′UTR is not required to silence dBigH1 expression in spermatogonia and, along the same lines, it is not sufficient to silence *vasa*-EGFP expression. Instead, in testes, dBigH1 silencing in spermatogonia is mediated by Bam [34]. Our results suggest that Bam is also required for dBigH1 silencing in cystocytes. However, in this case, its contribution might be indirect, through the activation of Brat expression [40]. Altogether these results suggest that, at least in part, the mechanisms governing translational regulation of dBigH1 expression are different in ovaries and testes.

We have also shown that Brat is required for dBigH1 silencing in gastrulated embryos. It has been reported that, during embryo development, Brat acts both as a translational repressor and a factor required for degradation/decay of maternal transcripts at MZT [58]. In this regard, sustained dBigH1 expression observed at gastrula stages in *brat* mutant embryos supports a contribution of Brat to *dBigH1* mRNA stability at MZT. Instead, in early oogenesis, Brat probably acts as a repressor of *dBigH1* mRNA translation since dBigH1 is silenced only transiently during TA divisions. Brat might also regulate the translation of maternal *dBigH1* transcripts in early embryogenesis. The factors that control Brat action in repression or mRNA destabilization remain to be determined.

Our results challenge the usually accepted view that germline-specific H1s replace somatic variants, which implies that their patterns of expression do not generally overlap. Instead, we have shown that somatic dH1 is broadly expressed during oogenesis and, though it ends up being replaced by dBigH1 in the oocyte, the two variants largely coexist except during TA divisions, where dH1 is expressed, but dBigH1 is not. A similar situation was reported in testes, where dH1 coexists with dBigH1 in GSCs and GBs, but it is the only variant expressed in TA divisions [34]. However, in this case, once proliferation stops, dH1 expression is strongly silenced in spermatocytes, while dBigH1 is highly expressed [34]. Later, dBigH1 is also silenced in spermatids [34]. Along the same lines, in *brat* mutant embryos, dBigH1 and dH1 also coexist at gastrula stages. Altogether, these results indicate that the patterns of expression of dH1 and dBigH1 are not necessarily exclusive. It is possible that dBigH1 and dH1 preferentially target different genomic loci since ectopic dBigH1 expression in S2 cells has shown that dBigH1 preferentially binds to and displaced dH1 from silent genomic regions with high dH1 content [71]. On the other hand, recent results suggest a more complex situation since, in null *bigH1* mutants generated by CRISPR/CAS9, the lack of maternal dBigH1 is compensated by the expression of somatic dH1 from the earliest stages of embryo development [43,72], suggesting that dBigH1 represses dH1 expression in the early *Drosophila* embryo. Further work is required to reach a better understanding of the actual link(s) between dBigH1 and dH1 expression and deposition.

Results reported here and elsewhere [19,34] show that the pattern of expression of dBigH1 is tightly regulated during germline lineage differentiation and embryogenesis, suggesting that dBigH1 plays specific functions in germline and embryo development. However, unveiling the functional contribution of dBigH1 is proving more difficult than anticipated. Based on defects associated with a genetic mutation generated through imperfect excision of a 5′UTR P-element insertion, dBigH1 was proposed to be essential during early embryogenesis, contributing to the activation of the zygotic genome [19]. In addition, RNAi-mediated depletion of dBigH1 in testes induced strong developmental defects and reduced fertility [34]. However, CRISPR/CAS9 null *bigH1* mutants turned out to be viable and fertile, progressing through embryogenesis likely due to the compensatory expression of somatic dH1 [43,72]. Moreover, CRISPR/CAS9 lines in which the CDS of dBigH1 was replaced by that of somatic dH1 are also viable, though showing DNA replication defects and altered chromatin condensation during early embryogenesis [73]. These observations suggest that to a large extent, dBigH1 and dH1 are functionally redundant, leaving the question of the possible specific functions of dBigH1 open.

In summary, our results show that the tumour suppressor Brat is crucial to silence dBigH1 expression in both ovaries and embryos. This regulatory mechanism might not be constrained to the germline. In this regard, it was reported that, while dBigH1 is not detected in the normal larval brain (or any other somatic tissue), it becomes ectopically expressed in some Brat-induced brain tumours [74]. To what extent dBigH1 expression contributes to malignant growth in somatic tissues remains to be determined. It also remains to be determined if Brat orthologues in other species (such as human TRIM2,3, which are implicated in malignant glioma [75]) have similar effects in the expression of embryonic H1 linker histones.

## Data accessibility

Data and materials are available from the authors upon reasonable request.
